# Identification of the nucleophile catalytic residue of GH51 α-l-arabinofuranosidase from *Pleurotus ostreatus*

**DOI:** 10.1186/s13568-015-0164-x

**Published:** 2015-12-21

**Authors:** Antonella Amore, Alfonso Iadonisi, Florence Vincent, Vincenza Faraco

**Affiliations:** Department of Chemical Sciences, University of Naples “Federico II”, Complesso Universitario Monte S. Angelo, via Cinthia, 4, 80126 Naples, Italy; CNRS, UMR7257, Aix-Marseille Universite, 163 Avenue de Luminy, Case 932, 13288 Marseille cedex 09, France; National Renewable Energy Laboratory, Biosciences Center, 15013 Denver West Parkway, Golden, CO 80401 USA

**Keywords:** Arabinofuranosidase, Lignocellulose, Site-directed mutagenesis

## Abstract

In this study, the recombinant α-l-arabinofuranosidase from the fungus *Pleurotus ostreatus* (rPoAbf) was subjected to site-directed mutagenesis in order to identify the catalytic nucleophile residue. Based on bioinformatics and homology modelling analyses, E449 was revealed to be the potential nucleophilic residue. Thus, the mutant E449G of PoAbf was recombinantly expressed in *Pichia pastoris* and its recombinant expression level and reactivity were investigated in comparison to the wild-type. The design of a suitable set of hydrolysis experiments in the presence or absence of alcoholic arabinosyl acceptors and/or formate salts allowed to unambiguously identify the residue E449 as the nucleophile residue involved in the retaining mechanism of this GH51 arabinofuranosidase. ^1^H NMR analysis was applied for the identification of the products and the assignement of their anomeric configuration.

## Introduction

α-l-arabinofuranosidases (α-l-AFases) (EC 3.2.1.55) take part in the hydrolysis of hemicelluloses, such as arabinoxylan, arabinogalactan, and l-arabinan, cleaving α-l-arabinofuranosidic linkages. As a consequence of their important role as a component of the enzymatic cocktail needed for lignocellulose hydrolysis (Marcolongo et al. 2014), the interest in the investigation of this class of enzymes is largely increasing.

Like other glycosyl hydrolases (GHs), α-l-arabinofuranosidases mediate glycosidic bond cleavage via acid/base-assisted catalysis, through either an inverting or a retaining mechanism. Retaining α-l-arabinofuranosidases are members of GH3, GH51 and GH54 families, that cleave the glycosidic bond using a two-step double-displacement mechanism. On the other hand, inverting α-l-arabinofuranosidases, belonging to GH43 family, use a single displacement mechanism (Shallom et al. 2002).

An α-l-arabinofuranosidase produced by *Pleurotus ostreatus* (PoAbf) during solid state fermentation on tomato pomace was identified, and the corresponding gene and cDNA were cloned and sequenced (Amore et al. 2012). The amino acid sequence similar to the other α-l-arabinofuranosidases indicated that the enzyme encoded by *poabf* can be classified as a family 51 glycoside hydrolase. Heterologous recombinant expression of PoAbf was carried out in *Pichia pastoris* and the recombinant enzyme (rPoAbf) was purified and characterized, revealing to be a versatile enzyme able to work on arabinooligosaccharides, with a higher affinity for the shorter ones, and on the natural polysaccharides linear arabinan and arabinoxylan, displaying both exo- and endoxylanase activities. It is worth noting that PoAbf shows very high stability in a broad range of pH. This enzyme was subjected to directed evolution experiments that allowed developing a mutant with improved activities (Giacobbe et al. 2014) and effect in biomass conversions (Marcolongo et al. 2014).

In this study, the catalytic mechanism of the enzyme PoAbf was elucidated and its nucleophile catalytic residue was investigated by bioinformatics and molecular modeling analyses. The catalytic nucleophile residue of PoAbf was identified by site-directed mutagenesis and analysis of reactivity of the mutant in comparison to the wild-type enzyme.

## Materials and methods

### Preparation and recombinant expression of the site-directed mutant E449G

The pPICZ-abf containing the cDNA encoding PoAbf (EMBL Data Library accession number HE565356) was used for recombinant expression in *P. pastoris* as previously reported (Amore et al. 2012). Site-directed mutagenesis was performed using the QuikChange site-directed mutagenesis kit (Stratagene, La Jolla, CA) and the pPICZ-abf as template. The mutagenic oligonucleotides adopted as primers are ACCTTCTACGAGGGAG**G**ATACGCCGCTATTAG (Fw) and CTAATAGCGGCGTAT**C**CTCCCTCGTAGAAGGT (Rev), with the mutated nucleotides underlined and bold.

The mutated gene was sequenced to confirm that only the desired mutations were inserted. The wild type and mutated proteins were overexpressed, purified and assayed as previously described (Amore et al. 2012).

α-l-Arabinofuranosidase activity was determined according to Yanay and Sato (2000). The activity was measured by spectrophotometric method with p-nitrophenyl α-l-arabinofuranoside (pNPA) (Gold Biotechnology, St Louis, MO, USA) as substrate, as previously described (Amore et al. 2012).

### Bioinformatic analysis and homology/molecular modeling

A multiple sequence alignment was performed with 50 GH51 sequences including PoAbf, and the glycosyl hydrolases (GH51) from *Leucoagaricus gongylophorus, Meripilus giganteus, Aspergillus niger, Leucoagaricus gongylophorus, Thermotoga maritima* (Tm-Afase), *Bacillus subtilis, Geobacillus stearohermophilus, Thermobacillus xylanilyticus* (Tx-Abf) and *Cellvibrio japonicus*, using MULTALIN (Corpet 1988). A 3D model of PoAbf was generated using MODELLER (Sali and Blundell, 1993; Söding et al. 2005) with default parameters and, as templates, the structures of the glycosyl hydrolases (GH51) from *Thermobacillus xylanilyticus* (PDB = 2VRQ) and *Thermotoga maritima* (PDB = 3UG3) (14 and 13 % sequence identity with PoAbf, respectively), selected using the TM-score from the HHpred server (http://toolkit.tuebingen.mpg.de/modeller).

### Stereochemical study of rPoAbf wt hydrolysis

**Fig. 1:**
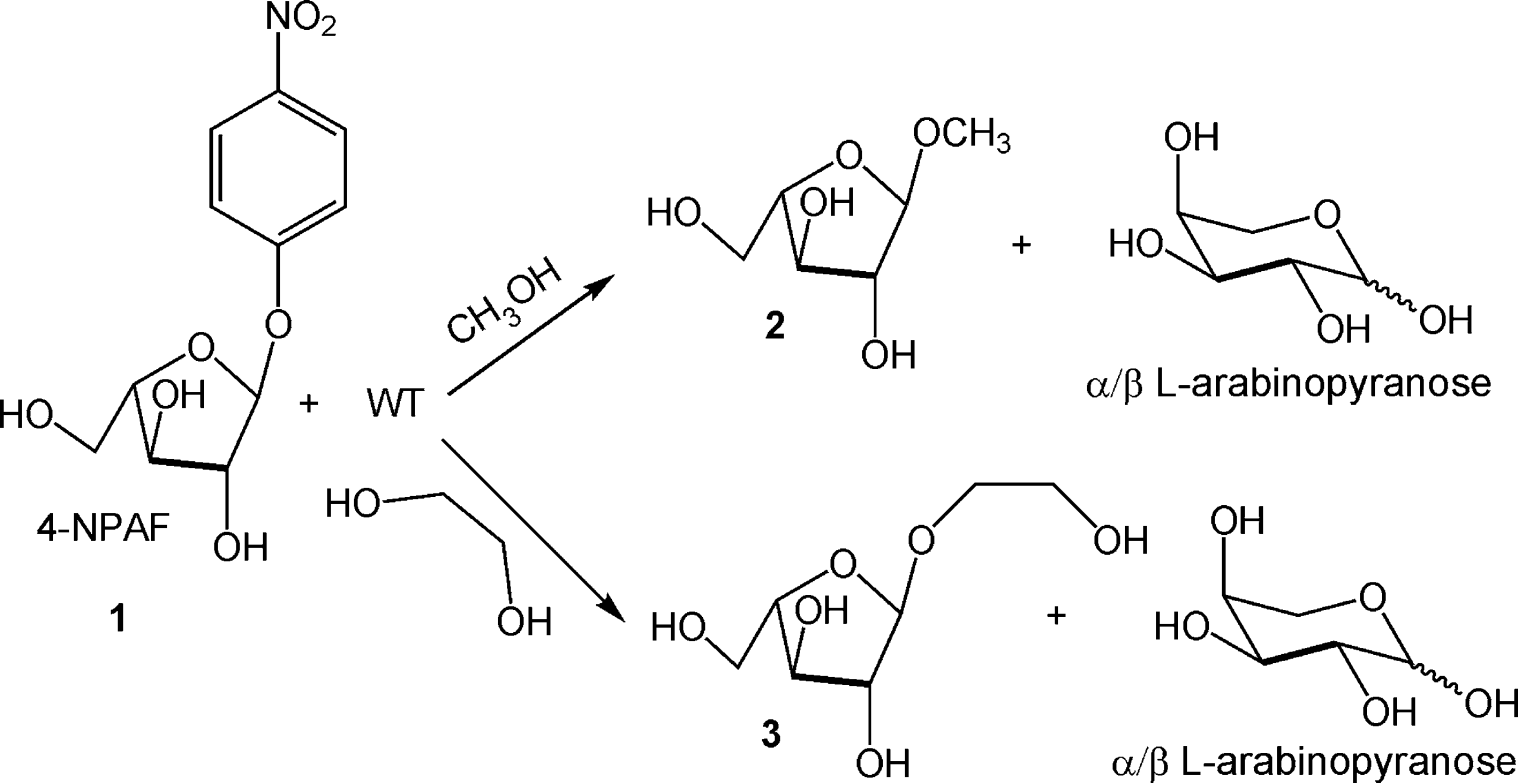
WT catalyzed a-arabinosylation of methanol or ethylene glycol

For the NMR characterization, the enzymatic reaction included wild-type PoAbf (0.2 mg), pNPA **1** (Fig. 1) (20 mg), and methanol (2.5 M) in a final volume of 5.5 mL. After 10 min at 40 °C, the reaction was quenched by the addition of 2 mM HgCl_2_ and lyophilized.

The reaction mixture was analyzed via TLC (eluent: ethyl acetate/methanol 9:1) and ^1^H NMR in D_2_O by comparison with authentic samples of l-arabinose (Sigma Aldrich, Milan, Italy) and methyl α-arabinofuranoside synthesized by following the procedure reported by van Der Klein et al. (1991).

### Identification of the reaction product of the nucleophile mutant

The investigation on the reactivity of the mutated enzyme was also performed by NMR analysis of the reaction mixtures. In this regard, a set of three alternative general conditions was designed for identifying the actual nucleophilic residue involved in the first step of the mechanism.

The first set of conditions entailed exposure of the mutated enzyme (375 μL, 1 mg/mL) to pNPA **1** (0.4 mg) (structure in Fig. 1) and a potential arabinosyl acceptor such as methanol or ethylene glycol (2.5 M), in a final volume of 1.5 mL.

A second set of conditions conditions entailed exposure of the mutated enzyme (375 μL, 1 mg/mL) to pNPA **1** (0.4 mg) and a potential external nucleophile such as sodium formate (2 M) in a final volume of 1.5 mL.

A third set of conditions entailed exposure of the mutated enzyme (375 μL, 1 mg/mL) to pNPA **1** (0.4 mg) in the presence of both ethylene glycol (2.5 M), as an arabinosyl acceptor, and sodium formate (2 M), as an external nucleophile.

Both the mutant and the wild-type samples were partially purified through 80 % ammonium sulfate precipitation, thus dialyzed and concentrated in a 10 kDa cut-off Centricon device (Millipore), in the presence of 50 mM sodium phosphate buffer, pH 6.5. All the reaction were carried out at 40 °C for 72 h.

**Fig. 2:**
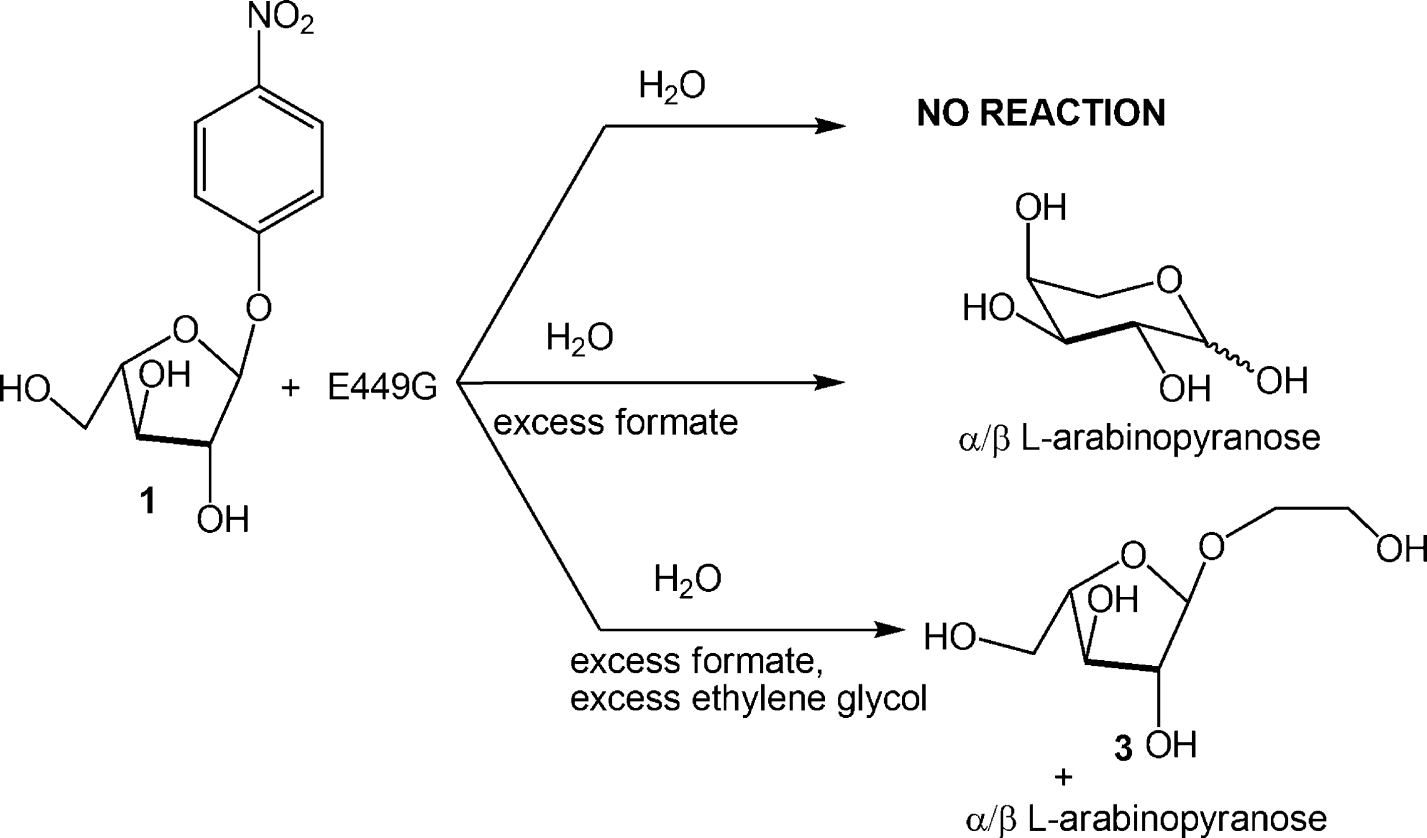
Summary of results of E449-catalyzed hydrolyses of **1** under differentiated conditions

Purification of α-arabinofuranoside **3** (Structure in Figs. 1, 2), derived from glycol ethylene, was performed by silica gel chromatography (eluent: ethyl acetate/methanol 9:1). Significant ^1^H NMR data (400 MHz, D_2_O) δ 5.10 (1H, d, J = 1.2 Hz, H-1), 4.16 (1H, dd, J = 1.2 and 3.6 Hz, H-2), 4.12 (1H, td, J = 3.6 and 6.0 Hz, H-4), 4.00 (1H, dd, J = 3.6 and 6.0 Hz, H-3), 3.90-3.65 (8H, overlapped signals, H_2_-5 and aglycon Hs).

## Results

### Identification of the reaction product of the wild type PoAbf

**Fig. 3:**
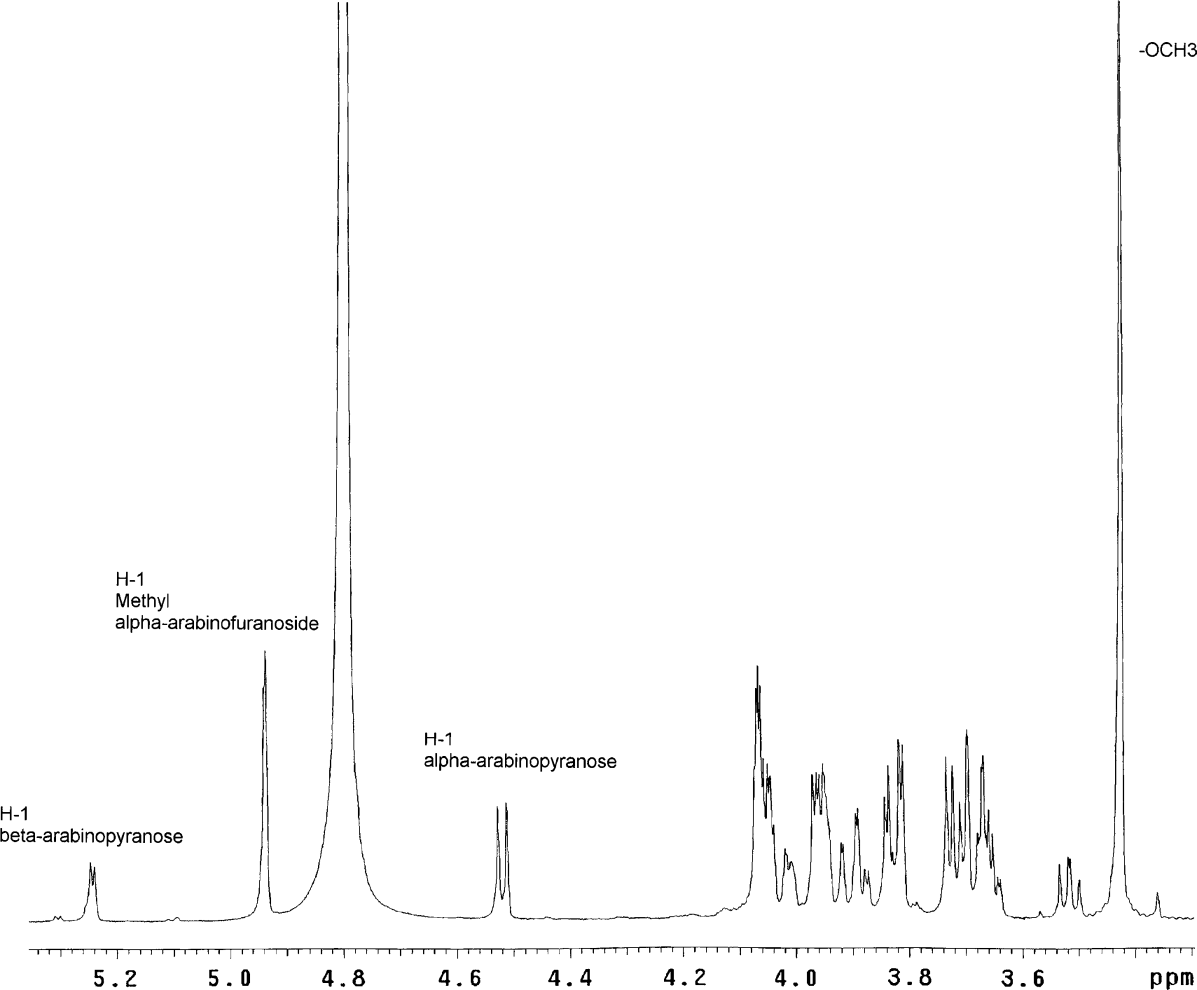
^1^H NMR of the crude mixture of the WT catalyzed hydrolysis of **1** in the presence of methanol

In this study, the investigation was initially aimed at elucidating the (retentive or inverting) mechanism of the hydrolysis reaction catalyzed by the arabinofuranosidase GH 51 PoAbf, performing the hydrolysis experiments in the presence of an excess of methanol or ethylene glycol (Fig. 1). The addiction of these arabinosyl acceptors was required due to the fact that the presence of water alone leads to the quick conversion of the expected arabinofuranose product into both its pyranose forms, with consequent loss of any stereochemical information on the process. Direct ^1^H NMR analysis in D_2_O of the water-soluble fraction from the lyophilized residue evidenced the presence of methyl α-arabinofuranoside **2** and l-arabinopyranose (as a mixture of α- and β-anomers), in an approximately equimolar amount. In Fig. 3, the ^1^H NMR spectrum of the mixture is reported, together with the attribution of the most significant signals.

The configuration of the glycosidic bond of methyl arabinofuranoside **2** was evidenced by the low J_1,2_ coupling constant (lower than 2 Hz) that is typical for α-arabinofuranosides (a value in the 4–5 Hz range is instead commonly found for β-anomers).

Under similar conditions (Fig. 1), ethylene glycol proved to be an even better acceptor than methanol, and also in this case an α-configured arabinofuranoside (**3**) was formed. Notably, product **3** largely prevailed in the crude mixture and the amount of free l-arabinose generated (as pyranose anomers) was sensibly reduced down to a 10–20 % yield.

Generation of α-arabinofuranosides with both methanol and ethylene glycol indicates the occurrence of a retaining mechanism with the nucleophilic attack of the alcohol acceptor (or water) to a β-configured glycosyl-enzyme intermediate formed in a previous step. The exclusive identification of l-arabinose in its pyranose forms also highlights the fast mutarotation of the initially generated α-arabinofuranose.

### Bioinformatic analysis and homology modelling

The sequence of PoAbf belongs to the GH51 enzyme family, identified in the Cazy (carbohydrate-active enzymes) database (Lombard et al. 2014). However, the analysis of the sequence of PoAbf revealed that it shows very low sequence identity (below 14 %) with many sequences from characterized GH51 enzymes, like *Thermotogs maritima* and *Thermobacillus xylanilyticus*. The sequence of PoAbf shows high sequence identity with 50 GH51 sequences from the public database, which together probably form a subfamily of GH51 enzymes distantly related to the main GH51 enzyme family.

**Fig. 4:**
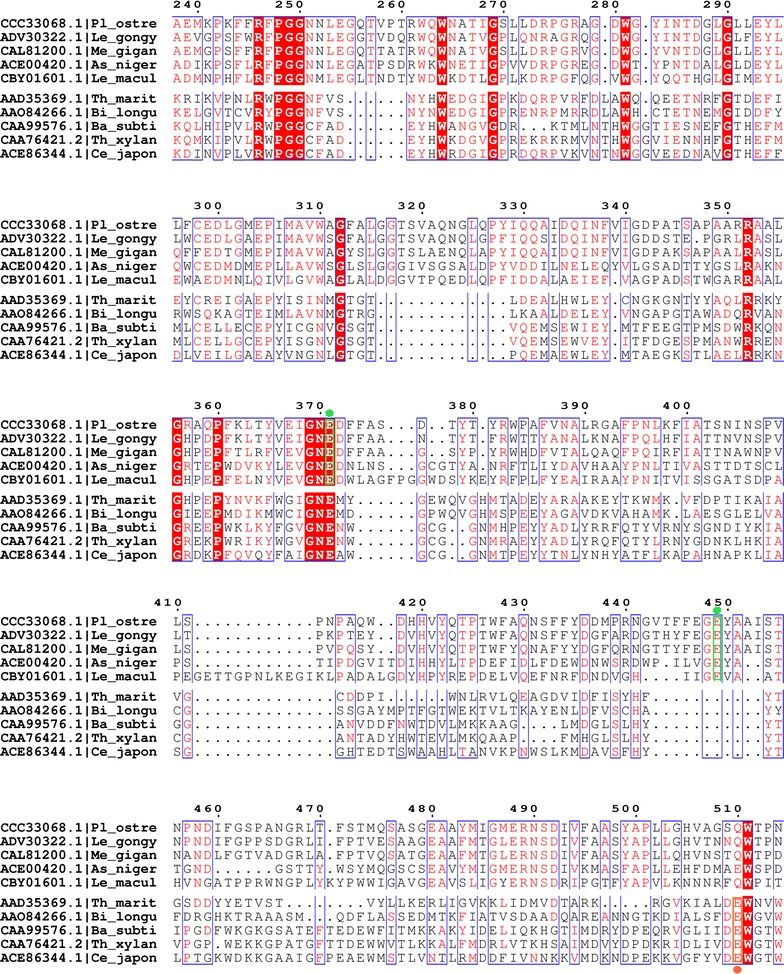
Sequence alignment of 10 GH51 sequences. Close up on sequence alignment with 4 sequences homologous to *Pleurotus ostreatus* namely, *Leucoagaricus gongylophorus*, *Meripilus giganteus*, *Aspergillus niger*, *Leucoagaricus gongylophorus*, and 5 bacterial GH51 sequences from characterized enzymes *Thermotoga maritima* (Tm-Afase), *Bacillus subtilis*, *Geobacillus stearohermophilus*, *Thermobacillus xylanilyticus* (Tx-Abf), *Cellvibrio japonicus*. The conserved general acid residue, corresponding to Glu172 in Tm-Afase and Glu176 in Tx-Abf, is conserved in Pleurotus ostreatus sequence and its homologs, and lies in position 371 (shown encased in *green* with a *green dot*). On the alignment we show the Glu nucleophile conserved in Pleurotus ostreatus and its homologs but not in the bacterial GH51 enzymes. The Glu nucleophile lies in position 449 and is shown encased in *green* with a *green* *dot*. The Glu nucleophile conserved among the GH51 enzyme sequences corresponding to E281 from Tm-AFase and E298 from Tx-Abf is shown encased in *orange* with an *orange dot*, and does not align with the conserved Glu nucleophile of Pleurotus ostreatus

**Fig. 5:**
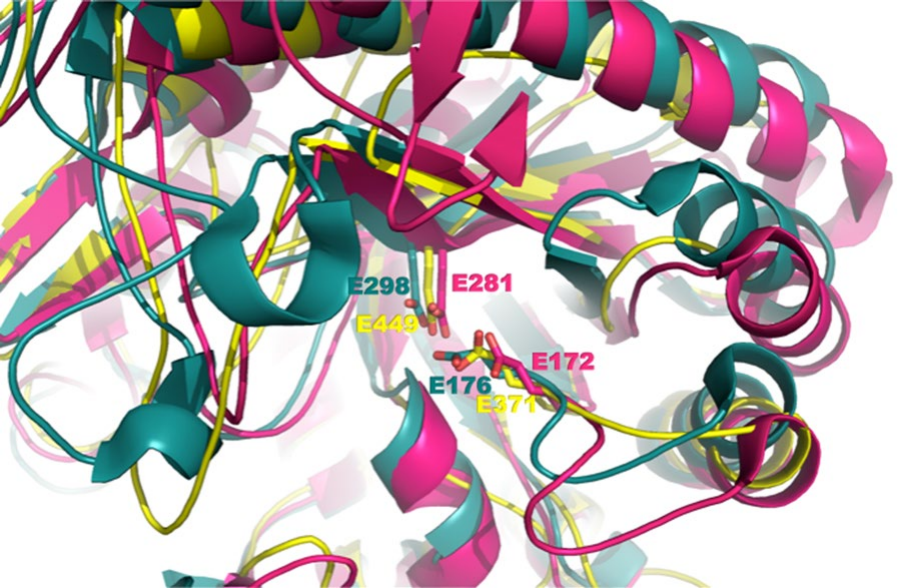
Structural model of PoAbf. Close up view of the overlay of the structural model of PoAbf (in *yellow*) with the structure of Tm-AFase (in *pink*) and Tx-Abf (in *blue*). This overlay shows the superimposition of the Glu acid/base residue 371 of *Pleurotus ostreatus* with the Glu acid/base from Tm-AFase (Glu 172) and Tx-Abf (Glu 176), as well as the Glu nucleophile on *Pleurotus ostreatus,* Glu 449, superimposing with Glu 281 from Tm-AFase and Glu 298 from Tx-Abf

In order to localise the catalytic residues on PoAbf sequence, 50 GH51 sequences homologous to PoAbf were aligned and revealed the strict conservation of E293, E371, E449 E480 and E488. As a second step, an alignment of PoAbf against all characterized GH51 (75 sequences) including *T. maritima* and *T. xylanilyticus* sequences was performed. This alignment identified the acid/base catalyst E371 on PoAbf, corresponding to the acid/base catalysts E172 and E176 on *T. maritima* Tm-AFase and *T. xylanyliticus* Tx-Abf, respectively (Fig. 4). However, we could not localise the nucelophile residue, as it was not superimposing with the nucleophile from the GH51 characterized enzymes. Therefore, in order to support the hypothesis that E371 is the acid/base catalyst and to localise the nucleophile residue, a homology model of PoAbf was generated using the Tm-AFase (PDB: 2VRQ) and Tx-Abf (PDB: 3UG3) structures as templates. The superimposition of PoAbf model on the structure of Tm-AFase and Tx-Abf confirmed the putative role of acid/base catalyst for E371 as it superimposes well with E172 and E176 from Tm-AFase and Tx-Abf, respectively. From this result, we could also determine the putative nucleophile residue, namely E449, as it superimposes with E281 from Tm-AFase and E298 from Tx-Abf (Fig. 5).

### Production and analysis of E449G PoAbf mutant

Recombinant expression of the mutant E449G of PoAbf was performed to investigate the potential role of E449 as nucleophile site. The production of arabinofuranosidase activity by E449G mutant of PoAbf was investigated in comparison to that of the wild type enzyme at 20 °C, since this had been demonstrated the optimal temperature for PoAbf production. The mutant E449G produced an activity level measured towards the pNP-arabinifuranoside tenfold lower than that evaluated for the wild-type (0.13 ± 0.01 versus 1.15 ± 0.01 U/mL). The activity was measured in the crude cuture broth, centrifuged after 6 days of growth.

It was demonstrated that the PoAbf mutant E449G displays a negligible hydrolysis on pNPA, either in the presence or in the absence of potential glycosyl acceptors such as methanol or ethylene glycol (Fig. 2). These results suggest that the mutated residue contains a functional group directly involved in the reaction mechanism.

Further experiments were performed by the addition of an excess of sodium formate to a mixture of pNPA and mutant E449, that restored the hydrolitic reactivity, even if at a much slower rate than PoAbf wild-type (Fig. 2). This result indicates that an exogenous carboxylate functionality could replace the carboxylate group belonging to the replaced glutamic acid residue. Unfortunately, NMR analysis was not capable of detecting the putative β-formyl arabinofuranoside intermediate, which is transiently formed in small amounts and it is also potentially amenable to a non-enzymatic degradation due to the potential occurrence of a fast internal trans-*O*-formylation process (Brecker et al. 2009).

In literature, it has been described that glycosidase mutants lacking the nucleophilic residue can be reactivated by addition of external formate salts, and this reactivation can be exploited for performing the glycosidation of appropriate acceptors properly added in the reaction mixture (Trincone et al. 2010). The term glycosynthase has been introduced to refer to such a kind of application. In an attempt to support the expected pivotal role played by the 449 residue, glycosynthase activity of mutant E449 was assessed by exposing pNPA to an excess of formate and an excess of ethylene glycol, the latter being the most effective α-arabinosyl acceptor according to the above described experiments on the PoAbf wild-type (Fig. 1). This experiment furnished a reaction mixture containing an approximately equimolar mixture of l-arabinose and ethylene glycol α- arabinofuranoside **3** (Fig. 2). The generation of the ethylene glycol α- arabinofuranoside represents a further evidence that formate can react as the replaced glutamic residue to give a β-configured intermediate wich in turn can react with ethylene glycol to yield the α-glycoside. As previously shown, under otherwise identical conditions but in the absence of formate, no appreciable hydrolytic event occurred (with recovery of pNPA **1**), and combination of all these results clearly points to a direct participation of formate in the reaction path and its stereocontrolled attack to pNPA to yield a β-configured intermediate.

## Discussion

The presence of a nucleophile amino acid is crucial in the GHs retaining mechanism, since it directly attacks the anomeric center to form a glycosyl–enzyme intermediate.

In this work, the E449 residue was shown to be the nucleophile residue involved in the reaction mechanism of the GH 51 α-l-arabinofurnaosidase from the fungus *Pleurotus ostreatus*.

The nucleophile E449 has been predicted based on the overlay of the crystal structures of Tm-AFase and Tx-Abf with a structure model of PoAbf. E449 was located in a position suitable to act as a putative nucleophile in PoAbf corresponding to the nucleophile E281 from Tm-AFase and E298 from Tx-Abf. Based on sequence similarities, it was not possible to predict unambiguously the nucleophile catalyst. Indeed the broad sequence diversity has limited the power of prediction and therefore the assignment of the catalytic residue. PoAbf belongs to a set of sequence distantly related to the characterized enzymes with 14 % sequence identity on average. Moreover, PoAbf has been shown to be a versatile enzyme able to work on different arabinooligosaccharides with exo and endo activities. PoAbf is also highly stable in a broad range of pH, which is not the case for most of the other α-l-arabinofuranosidases (Amore et al. 2012). It is possible that the set of sequences including PoAbf, represent a new subfamily in the GH51 family. Subfamily classification of GH13, GH30 and GH5 has demonstrated that the majority of the defined subfamilies were monospecific, thus indicating a significantly better correlation of substrate specificity between sequences at the subfamily level than at the family level (Stam et al. 2006; St John et al. 2010; Aspeborg et al. 2012). For GH73 family, the catalytic mechanism and the ligand specificity seem to correlate the sequences in each subfamily (Lipski et al. 2015). Therefore, the large substrate specifities of PoAbf, or its dual catalytic activity could illustrate the subfamily it belongs to. A thorough bioinformatic analysis conducted on this broad set of sequences should allow the identification of yet uncharacterized subfamilies.

The activity of the inactive mutant E449G was restored by the addiction of external nucleophile, such as sodium formate, using an approach similar to the one followed by Moracci et al. (1998) who studied the effect of external nucleophiles on the mechanism of action of a hyperthermophilic *β*-Glycosidase from *Sulfolobus solfataricus.* As far as the study of other arabinofuranosidases belonging to the retaining GH51 family is concerned, Shallom et al. (2002) have studied for the first time the catalytic properties of a *Geobacillus stearothermophilus* T6 α-l-arabinofuranosidase, with the identification of its catalytic residues: Glu175, the acid/base, and Glu294, the nucleophile. The arabinofuranosidase from *Therobacillus xylanilyticus* (Tx-Abf) has been shown to be a retaining enzyme that catalyzes the hydrolysis of glycosidic bonds through a double displacement mechanism, with Glu176 as the acid/base and Glu298 as the nucleophile (Debeche et al. 2002).

With regards to other glycosyl hydrolase families, Viladot et al. (2001) studied the hydrolytic activity of the nucleophile-less E134A mutant 1,3-1,4-β-glucanase, showing that its activity was restored by exogenous formate. Other retaining glycosidases mutated at the catalytic nucleophile have been reported to be rescued by formate, such as the *Agrobacteriumfaecalis* β-glucosidase (Wang et al. 1994) and the *Cellulomonasfimi* exo-β-1,4-glycanase (Macleod et al. 1996).

The GH51 arabinofuranosidase from *Pseudomonas cellulosa* was investigated by Beylot et al. (2001) who designed and characterized the mutants E194A and E321A, which were previously defined to be the catalytic acid\base and nucleophile residues, respectively. Beylot et al. were unsuccessful in reactivating the E194A and E321A mutants with azide, since the ions could not penetrate the pocket active site (typical of glycoside hydrolases that remove terminal sugars from polysaccharides) of the enzyme when occupied by the substrate. However, based on the biochemical characterization of the mutants, they confirmed Glu-194 and Glu-321 to be part of the key catalytic residues of this GH 51 arabinofurnaosidase.

Structural studies of the GH51 from *Thermobacillus xylanilyticus* were performed to asses the role of the residues H98 and W99 belonging to the 39-residue-long β2a2 loop (P71–G109), whose flexibility allows the operational activation of the active site (Arab-Jaziri et al. 2012). In particular, substitution of H98 and W99 by alanine or phenylalanine, negatively affected K_M_ and⁄or k_cat_. Souza et al. (2011) have structurally characterized the GH51 TpAraF from *Thermotoga petrophila* RKU-1 using X-ray crystallography, small angle X-ray scattering (SAXS) and spectroscopy. They provided data regarding protein stability, oligomerization, and described an unique motif in TpAraF, which modifies the active-site pocket. In particular, they defined H77, E99, D132, N146, Y148, E176, H181, E186, R189, K192, E193, K196, E255, K261, K262, and K363 as the key residues involved in GH51 TpAraF hexameric structure stabilization.

In view of the GHs improvement by protein engineering, many papers concerning the study of their active site residues have been recently published

The very highly conserved residues D142, D144 and E146 in *Manduca sexta* (tobacco hornworm) chitinase, were investigated as potential active site residues, using site-directed mutagenesis for their substitutions with other amino acids (Lu et al. 2002). Collins et al. (2005) studied the active site residues of a glycoside hydrolase family 8 xylanase, delineating the crucial role played by each active site residues. The results achieved by Oh et al. (2008) demonstrated that H119 is the critical residue in the active site of *Thermuscaldophilus* GK24 b-glycosidase. An approach based on the site directed mutagenesis was followed, with the preparation of two mutants showing a total loss of activity. Dodd et al. 2010, provided insight into the role of the active site residues of a GH3 xylanase from the rumen bacterium *Prevotella bryantii* B14. Particularly, their results support the hypothesis that Arg177, Lys214, and His215 form a highly conserved, core set of charged amino acid residues in GH3 enzymes, involved in the formation of linkages with the substrate. More recently, in order to get insight into the molecular basis of the substrate specificity of CpMan5B, a glycoside hydrolase (GH) family 5 enzyme exhibiting both β-1,4-mannosidic and β-1,4-glucosidic cleavage activities, Oyama et al. (2013) solved the crystal structure of the protein. The crystal structure of CpMan5B and the comparisons to the crystal structure of other mannanases (CtCel5C, subfamily GH5_37; TmCel5A, subfamily GH5_25) revealed important differences in several active site residues (Y12, H84, N92, N136, and R196). Thirteen mutant proteins were produced, in order to evaluate the residues role in enzyme function. The R196A mutation eliminated all the detectable activity, indicating that this residue is essential for catalysis.

The findings reported in this paper provide further evidence of the covalent nature of the glycosyl-enzyme intermediate produced in the reaction mechanism of GH 51 arabinofuranosidase, highlighting the crucial role of the nucleophilic residues in the reaction mechanism of retaining glycosidases.
